# Middle-Upper Pleistocene climate changes shaped the divergence and demography of *Cycas guizhouensis* (Cycadaceae): Evidence from DNA sequences and microsatellite markers

**DOI:** 10.1038/srep27368

**Published:** 2016-06-07

**Authors:** Xiuyan Feng, Ying Zheng, Xun Gong

**Affiliations:** 1 Key Laboratory for Plant Diversity and Biogeography of East Asia, Kunming Institute of Botany, Chinese Academy of Sciences, Kunming 650201, Yunnan, China; 2 University of Chinese Academy of Sciences, Beijing 100049, China

## Abstract

Climatic oscillations in the Pleistocene have had profound effects on the demography and genetic diversity of many extant species. *Cycas guizhouensis* Lan & R.F. Zou is an endemic and endangered species in Southwest China that is primarily distributed along the valleys of the Nanpan River. In this study, we used four chloroplast DNAs (cpDNA), three nuclear genes (nDNA) and 13 microsatellite (SSR) loci to investigate the genetic structure, divergence time and demographic history of 11 populations of *C. guizhouensis*. High genetic diversity and high levels of genetic differentiation among the populations were observed. Two evolutionary units were revealed based on network and Structure analysis. The divergence time estimations suggested that haplotypes of *C. guizhouensis* were diverged during the Middle-Upper Pleistocene. Additionally, the demographic histories deduced from different DNA sequences were discordant, but overall indicated that *C. guizhouensis* had experienced a recent population expansion during the post-glacial period. Microsatellite data revealed that there was a contraction in effective population size in the past. These genetic features allow conservation measures to be taken to ensure the protection of this endangered species from extinction.

Different regions have experienced different scales and frequencies of glaciations in the Pleistocene^1^. Although China was not glaciated during the Pleistocene^2^ and had a relatively mild Quaternary climate^3^, climatic oscillations in the Pleistocene have also had profound effects on the demography and genetic diversity of many extant species^4^,^5^,^6^,^7^. To respond to climatic oscillations, some species contracted into glacial refugia, became extinct and possibly locally adapted^6^,^8^. Nevertheless, historical processes leave imprints on the genetic structure of existing populations, especially those of long-lived and sessile organisms^9^. As an ancient and perennial gymnosperm, cycads play an important role for tracking the evolutionary history of seed plants. An increasing number of studies are examining the evolutionary history of cycads and how they have responded to historic climate change in recent years^9^,^10^,^11^.

Cycads are generally considered to be the oldest group of living seed plants. Cycads reached their greatest levels of diversity during the Jurassic-Cretaceous (approximately 199.6 to 65.5 million years ago, MYA). Their diversity dwindled to the present number of species (approximately 300 species) as a result of the emergence and dominance of flowering plants^12^,^13^,^14^,^15^,^16^. Although the cycad lineage is ancient, research based on a fossil-calibrated molecular phylogeny showed that cycads underwent a recent synchronous global rediversification in the late Miocene, indicating that extant cycad species are not much older than 12 million years^14^. Currently, cycads contain two families (Cycadaceae, Zamiaceae) with ten genera, primarily distributed in Africa, Asia, Australia and South and Central America^17^. Unfortunately, cycad species are threatened with extinction because of over exploitation and habitat loss. In China, there is only one cycad genus, *Cycas*, belonging to Cycadaceae^18^. All of the *Cycas* species are given ‘First Grade’ conservation status in China^19^.

Recent research which has combined ancestral area reconstructions with fossil evidence proposed that the origin of *Cycas* was in South China, with the deepest divergence occurring via a vicariant barrier: the Red River Fault^20^. In South China, *Cycas* species generally grow on low-altitude slopes of ridges and cliffs along river valleys. Their fertile seeds are large, heavy and sink in water, which precludes water dispersal over long distances. In addition, cycad seeds contain virulent cycasin, preventing their dispersal by animals for long distances. Thus, in this region, seed dispersal was likely limited to short distances^21^,^22^,^23^, resulting in the expected high levels of genetic differentiation as observed in maternally inherited DNA. *Cycas* species are dioecious and allogamous. In plant species, both the mating system and dispersal mechanism have important effects on genetic differentiation among populations^24^,^25^. For neutral markers, genetic differentiation is typically influenced by genetic drift, mutation of novel alleles and gene flow between populations^26^. If levels of gene flow between populations are sufficient, there may be no relationship between geographic distance and genetic differentiation.

*Cycas guizhouensis* Lan & R.F. Zou was described in 1983 and can be identified by its morphological characteristics of a cylindrical trunk up to one meter in height, fusiform male cone, loose and open female cone, densely hairy sporophylls, nearly round apical lobe (the margins with numerous tapered lobes) and yellow with reddish brown mucro of seeds (nearly globose)^13^. *Cycas guizhouensis* is endemic to Southwest China, occurring primarily in the valleys of the Nanpan River basin of southwestern Guizhou, northwestern Guangxi and the eastern Yunnan provinces. The species mainly prefers sand or limestone habitats where the climate is characterized by mild with cool, moist summers and moist, frost-free winters^13^. In recent decades, *C. guizhouensis* has been severely threatened by rapid habitat destruction for the cultivation of economic plants and over-collection because of its edible stem and ornamental attributes. This species has dramatically decreased in numbers and is becoming endangered. Field surveys have shown that there are two populations with fewer than 20 individuals. Thus, the need to develop effective protection measures for this species is urgent.

Understanding the genetic variation, genetic structure and demographic history in a rare species is essential for the establishment of effective and efficient conservation practices. Protection measures can be proposed by relying on detailed population genetic studies. Several population genetic studies on cycads based on different molecular markers have been recently published^9^,^10^,^11^,^19^,^27^,^28^. These publications provide references for the study of other cycad species. Previous studies on the genetic diversity of *C. guizhouensis* were by Yang and Meerow who used allozymes to investigate three populations of *C. guizhouensis*, and by Xiao who studied 12 populations of *C. guizhouensis* using ISSR markers^19^,^29^. These two studies were not consistent with each other in the levels of genetic differentiation reported between populations. Meanwhile, both of these markers in previous studies have their shortcomings: the number of allozyme loci that can be probed is limited and allozyme variation may not be able to accurately or completely measure nucleotide variation in the genome^30^. ISSR markers are dominant and are not able to distinguish homozygosity and heterozygosity directly; in addition, errors in counting bands may also arise. However, the nuclear DNA (nDNA) of cycads is biparentally inherited and is dispersed by both seeds and pollen. The organelle DNA (cpDNA) is maternally inherited and is dispersed only through seeds^31^. Microsatellite markers (SSRs) are known to be codominant and possess more genetic variation than other molecular markers. To comprehensively investigate genetic variation in *C. guizhouensis* and compare these results with previous studies on this species, we used four cpDNAs (*psb*A-*trn*H^32^, *psb*M-*trn*D^33^, *trn*L-*trn*T^34^ and *trn*L-*trn*F^34^), three nDNAs (*GTP*, GTP genes^35^; *PHYP*, phytochrome P gene and *F3H*, flavanone 3-hydroxyrase gene (unpublished)) and 13 microsatellite markers^36^,^37^,^38^,^39^,^40^,^41^,^42^ in this study, aiming to estimate the genetic variation, genetic structure and demographic history of *C. guizhouensis* in an further effort to investigate the causes of its endangerment and provide basic guidelines for its conservation.

## Results

### DNA sequence diversity and haplotype fixation and distribution

**Figure 1:**
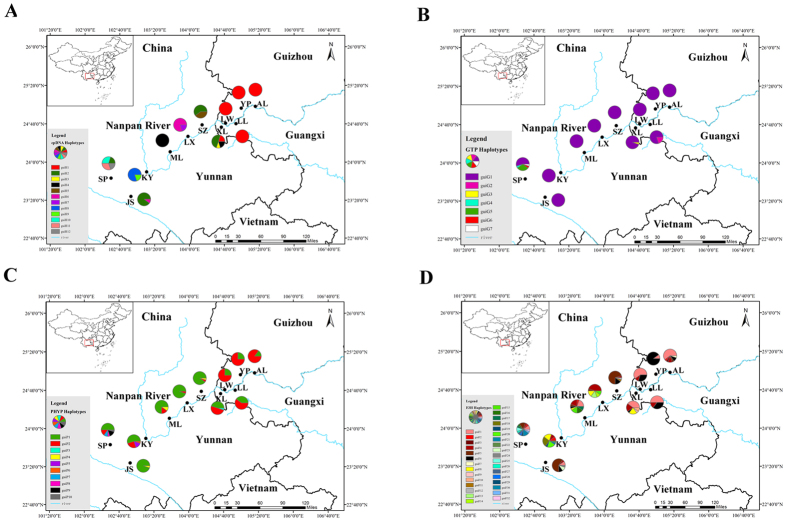
Geographical distribution of 11 populations of *C. guizhouensis* and distribution of its haplotypes detected from cpDNA (**A**), *GTP* (**B**), *PHYP* (**C**) and *F3H* (**D**). Population codes refer to Table S1. Maps were drawn using the software ArcGIS version 10.2 (http://desktop.arcgis.com) and modified using Photoshop (Adobe Corporation, California, America).

The four cpDNA regions surveyed across the 110 individuals (11 populations, Table S1) of *C. guizhouensis* identified 12 haplotypes (guiH1-guiH12) in total. Of these, eight haplotypes were specific to one population, whereas haplotypes guiH1, guiH2, guiH4 and guiH6 were shared by five, four, two and two populations, respectively. Detection of recombination in nuclear genes showed that no recombination occurred in *GTP* and *PHYP*. In contrast, eight recombination events were occurred in *F3H*. Most software used in our analysis didn’t need the data to remove recombination. Thus, we did not remove recombination from gene *F3H* because some individuals with recombination were also removed. A total of seven haplotypes (guiG1-guiG7) were derived from the nuclear gene *GTP*, of which haplotype guiG1 was the most abundant and predominant in all 11 populations. The remaining six haplotypes were private. Ten haplotypes (guiP1-guiP10) were derived from the nuclear gene *PHYP*. Of these, haplotypes guiP1 and guiP2 were most frequent and were widely shared by all 11 and ten populations, respectively. Haplotype guiP4 was distributed in three populations and the remaining haplotypes were specific to single populations. The nuclear gene *F3H* identified 32 haplotypes (guiF1-guiF32) in total. guiF1 and guiF4 were the most widely distributed haplotypes. Information on cpDNA and three nDNA haplotypes and their distribution in populations are shown in Table S2 and Fig. 1, respectively.

Genetic diversity indices *H*d and *P*i for each population were summarized in Table S2 and were highly variable among cpDNA and nDNA. In sum, population SP had the highest genetic diversity. Total genetic diversity *H*_T_ was higher than the average intrapopulation diversity *H*s (Table S3), which meant that there were high levels of genetic differentiation (*N*_ST_, ranging from 0.192 to 0.698; *G*_ST_, ranging from 0.233 to 0.676). *N*_ST_ was not significantly greater than *G*_ST_ (*P* > 0.05), suggesting that there was no correspondence between haplotype similarities and their geographic distribution in *C. guizhouensis*.

The AMOVA revealed that there was more variation (69.82%) partitioned among populations rather than within (30.18%) populations based on cpDNA data, whereas there was less variation (24.11%, 30.75% and 19.20%) partitioned among populations than variation (75.89%, 69.25% and 80.80%) within populations based on nuclear genes *GTP, PHYP*, and *F3H*, respectively. *F*_ST_ for these genes thus ranged from 0.192–0.698^43^, with all indicating highly significant genetic differentiation among *C. guizhouensis* populations (Table 1).

### Phylogeny and network of haplotypes

Phylogenetic relationships among cpDNA and nDNA haplotypes were constructed using Bayesian inference (Fig. S1). The four phylogenies were strongly supported with Bayesian probabilities, revealing a haplotype monophyly for *C. guizhouensis* with *Cycas edentat*a and *Cycas rumphii* as outgroups. The 12 cpDNA haplotypes were grouped into two clades, showing that haplotype guiH7 was the firstly diverged haplotype, and the others were grouped into a clade. Among this clade, haplotypes guiH2, guiH3, guiH4, guiH5, guiH8, guiH9 and guiH10 were clustered into a subclade, indicating that they were more closely related than others, although the phylogenetic relationship within the subclade was not resolved (Fig. S1A). The *GTP* haplotype phylogenetic tree showed that haplotypes guiG1, guiG2 and guiG4 were grouped together, and haplotypes guiG6 and guiG7 were clustered together, indicating that they shared closer relationships, respectively (Fig. S1B). For the *PHYP* haplotypes in the Bayesian phylogram, guiP2, guiP3, guiP7 were firstly and parallelly diverged haplotypes, while the relationship of the remaining seven haplotypes which formed a subclade was not resolved, either (Fig. S1C). Recombination gave rise to many haplotypes in the nuclear gene *F3H*. For haplotypes guiF13, guiF14 and guiF29 that were the earliest diverged in the *F3H* phylogram, their further relationships had not been figured out for lacking sufficient informative sites. Within the subsequent diverged but weakly supported clade (PP = 57) where 26 haplotypes were clustered in, although some genetic relationships of some *F3H* haplotypes were basically resolved such as haplotypes guiF1, guiF5 and guiF10, parallel relationships were also observed in most haplotypes (Fig. S1D).

**Figure 2:**
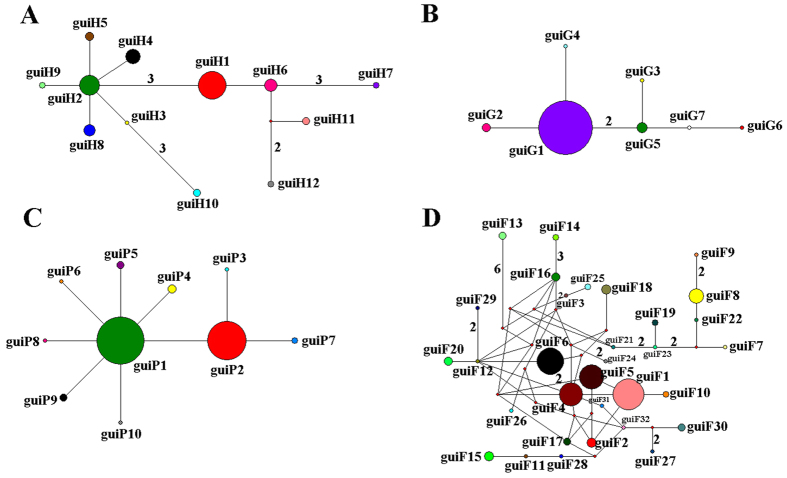
Network of haplotypes of *C. guizhouensis* based on cpDNA (**A**), *GTP* (**B**), *PHYP* (**C**) and *F3H* (**D**). The numbers on branches indicate mutational steps. Haplotype distribution in 11 populations refers to Table S2.

Genealogies reflecting topology and haplotype frequency were constructed for cpDNA and three nuclear genes (Fig. 2). The haplotype network of cpDNA was concordant with the haplotype network of nuclear genes *GTP* and *PHYP*, with each showing 1–2 centrally located nodes representing hypothetical ancestral haplotypes (Fig. 2A–C). The remaining haplotypes were linked to these central haplotypes by 1–3 steps in a star-like network. A reticulate evolutionary relationship was observed in the *F3H* haplotype network (Fig. 2D).

### Phylogenetic divergence time estimation from haplotypes

**Figure 3:**
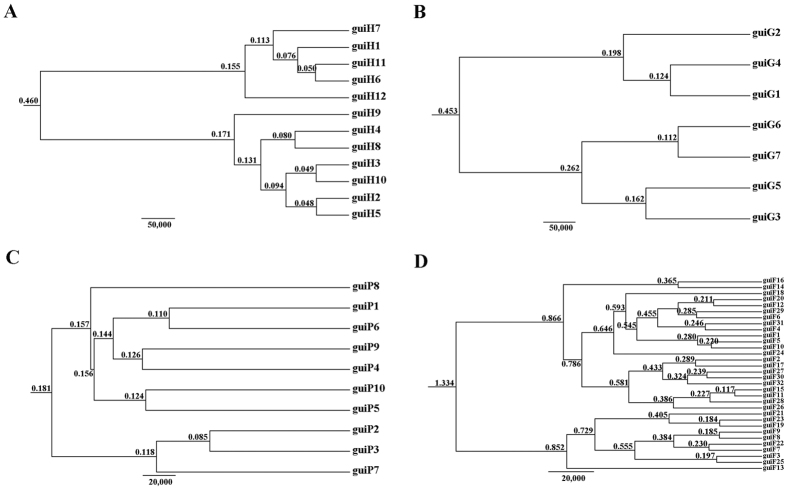
BEAST-derived trees based on cpDNA (**A**) and the nuclear genes *GTP* (**B**), *PHYP* (**C**) and *F3H* (**D**). The numbers on branches represent divergence time (MYA). Haplotype distribution in 11 populations refers to Table S2.

The BEAST-derived trees for the four DNA sequences revealed similar topologies (Fig. 3). The 12 cpDNA haplotypes clustered into two lineages, one including five haplotypes with guiH1 as the ancestral haplotype and the other including seven haplotypes with guiH2 as the ancestral haplotype. The two lineages were split at approximately 0.460 MYA and haplotypes in the inner lineages were diverged from 0.155 MYA to 0.050 MYA and 0.171 MYA to 0.048 MYA, respectively (Fig. 3A). For the three nDNA haplotype trees, haplotypes were also grouped into two lineages, separately coalescing at 0.453 MYA (*GTP*), 0.181 MYA (*PHYP*) and 1.334 MYA (*F3H*) (Fig. 3B–D). Coalescent events occurred much earlier in *F3H* than in *GTP, PHYP* and cpDNA. In total, most tip haplotypes in the four BEAST-derived trees diverged recently from their common ancestors. These results entirely implied that haplotypes of *C. guizhouensis* diverged in the Middle-Upper Pleistocene.

### Neutrality test, mismatch analysis and the Bayesian Skyline Plot

**Figure 4:**
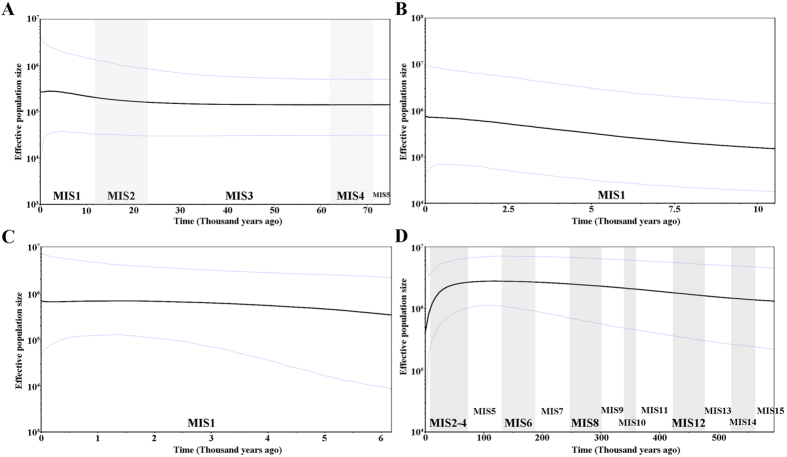
Bayesian skyline plots based on cpDNA (**A**) and the nuclear genes *GTP* (**B**), *PHYP* (**C**) and *F3H* (**D**) for the estimate of fluctuations in effective population size over time. Black line: median estimation; area between gray lines: 95% confidence interval. MIS: Marine Isotope Stage.

Values for Tajima’s *D*, Fu and Li’s D^*^ and F^*^, Fu’s *F*s tests as well as SSD and raggedness statistics were listed in Table S4. For cpDNA, all of the values were positive and non-significant, indicating that *C. guizhouensis* had not undergone a recent population expansion. For the nuclear genes, all values were non-significant except for significant negative values of Fu’s *F*s for all three genes, which suggested the presence of population growth or recombination^44^. Mismatch distribution analyses displayed bimodal and multimodal graphs for cpDNA and *F3H*, respectively (Fig. S2A, D), but unimodal graphs for *GTP* and *PHYP* (Fig. S2B, C). A multimodal curve is indicative of a population at demographic equilibrium, whereas a unimodal curve is indicative of recent population expansion. More detailed evidence of demographic came from the Bayesian Skyline Plots. Based on the pattern of variation in cpDNA, *C. guizhouensis* had a long history of a constant population size, followed by a slight population expansion (over 25-4 Ka years) and a subsequent slight decline (approximately 2 Ka-present) (Fig. 4A). During the recent 10 Ka years, rapid population growth was detected in *C. guizhouensis* based on the gene *GTP* (Fig. 4B), while slow population growth with subsequent slight declines in demography was detected in the Bayesian Skyline Plot of the gene *PHYP* (Fig. 4C). In contrast, coalescence of the gene *F3H* dated back to much longer ago, showing that *C. guizhouensis* experienced a long period of continuous growth over 600–80 Ka years before undergoing a decline from approximately 50 Ka years ago to the present (Fig. 4D).

### Nuclear microsatellite genotyping

Diversity estimates from 13 microsatellites varied among populations (Table 2), with the lowest and highest measures of diversity consistently found in populations YP and SP, respectively. Fixation indices (*F*) were positive for all populations, with a mean value *F* = 0.204, indicating that there was a high level of inbreeding within each population. This inbreeding resulted in a deficiency of heterozygotes and significant deviations from Hardy-Weinberg equilibrium at most loci and in most populations (Table S5). Estimates of effective population sizes (Ne) with the lowest allele frequency (0.05) were listed in Table 2. There were only two populations, LL and SP, whose Nes were greater than 50. The AMOVA revealed that 13.80% of the genetic variation was partitioned among populations and 86.20% was partitioned within populations with the significant genetic differentiation coefficient *F*_ST_ = 0.138 (Table 1). The correlation between genetic and geographic distances was not significant (P = 0.18), indicating that there was no significant effect of isolation by distance (IBD) (Fig. S3). Estimates of gene flow (*N*m) between pairs of populations were shown in Table S6. Most of these estimates was greater than 1.

**Figure 5:**
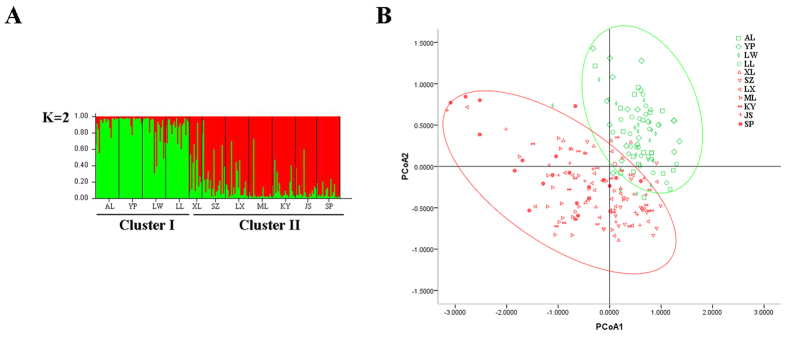
(**A**) Bayesian inference using STRUCTURE (K = 2) and (**B**) Principal coordinate analysis (PCoA) of SSR phenotype from 11 populations of 209 individuals of *C. guizhouensis*.

The clustering analyses resulted in population groupings that were generally consistent with the above haplotype aggregation analyses. The UPGMA clustering dendrogram, Structure analysis, and principal coordinate analysis (PCoA), all indicated the same general grouping: individuals belonging to populations AL, YP, LW and LL clustered into one group (Clade I), while the remaining individuals clustered into a second group (Clade II) (Fig. S4; Fig. 5).

The bottleneck analysis showed that no populations had a significant excess of heterozygosity in the two methods under two models, indicating that none of them deviated from mutation-drift equilibrium (Table 3). Mode shift models showed that all of the populations had a normal L-shaped distribution, which suggested that *C. guizhouensis* had not experienced a recent severe bottleneck. However, *C. guizhouensis* apparently experienced a population decline (bottleneck) in history as inferred from the calculation of Garza-Williamson indices which were lower than the critical *M*c value of 0.68 (Table 3).

## Discussion

Previous study on genetic patterns of *C. guizhouensis* using ISSR markers showed that this species has low genetic diversity and a high degree of differentiation among populations with the highest genetic diversity in population JS and the lowest in SZ^19^. In contrast, via DNA sequences (Tables 1, S2 and S3) and microsatellites (Tables 1 and 2) data analysis, our study revealed that *C. guizhouensis* had high genetic diversity and high genetic differentiation, respectively. However, population SP had the highest genetic diversity and YP had the lowest (Tables S2 and 2). Our results were not completely consistent with the previous study^19^. The types of molecular markers, the number of populations and sample sizes per population, life history traits and distance between populations can all affect genetic variation estimates within or among species^45^. Most *Cycas* species in Asia are narrowly distributed and their life history traits, such as dioecy, longer life span and pollination syndromes, are similar^46^. Thus, we can compare the genetic diversity of *C. guizhouensis* with other Asian *Cycas* species using the similar molecular markers. *Cycas guizhouensis* had higher genetic diversity (Tables 2, S2 and S3) than *C. debaoensis*^27^, but had slightly lower genetic diversity than *C. simplicipnna*^9^ and *C. multipinnata*^10^. cpDNA-based studies on 170 plant species revealed a mean value of *H*_T_ = 0.67^47^, while *H*_T_ estimated from *C. guizhouensis* by four cpDNA was 0.841 which implied that *C. guizhouensis* had a high level of genetic diversity.

All 11 *C. guizhouensis* populations deviated significantly from Hardy-Weinberg equilibrium (Table S5), and the fixation indices (*F*) were also all greater than zero (Table 2). Therefore, we can conclude that *C. guizhouensis* populations are notably deficient in heterozygosity and have experienced severe levels of inbreeding, which have contributed to a high level of genetic differentiation. In general, mating systems and dispersal mechanisms have significant effects on levels of genetic differentiation observed among populations of plant species. *Cycas guizhouensis* is dioecious with both wind and insects as primary pollen dispersal vectors^23^. This species has evolved large, heavy seeds that sink, limiting their dispersal by water^21^. These seeds also contain the neurotoxin cycasin, which limits the ability of animals to disperse them over long distances^23^. Most seeds germinate and grow near the mother plant, increasing the chances of inbreeding within the species. Consistent with restricted seed dispersal^21^,^23^, cpDNA data showed a high level of genetic differentiation and restricted seed-mediated gene flow among populations, despite their relative proximity. In contrast, our microsatellite data indicate much higher rates of interpopulation gene flow at nuclear loci with *N*m estimates mostly greater than 1 (Table S6). If *N*m > 1, gene flow can largely prevent population differentiation due to genetic drift, while if *N*m < 1, high levels of differentiation will develop^48^.

The AMOVA results assessed from cpDNA data revealed that more genetic variation existed among populations and less within populations, while the opposite result was obtained from nuclear genes and microsatellites (Table 1). This inconsistency can be explained by the different inheritance patterns and evolutionary rates of organelle DNA and nuclear DNA. In cycads, chloroplast DNA (cpDNA) is haploid, maternally inherited, and slowly evolving, while nuclear DNA (nDNA) is diploid, biparentally inherited, and more rapidly evolving^31^. These factors combined with typically greater rates of pollen- than seed-mediated gene flow^25^,^47^ contribute to the maintenance of higher within- and lower between-population nuclear than chloroplast diversity.

The observation of isolation by distance (IBD) was generally consistent with the previous study^19^, showing that there was no significant correlation between nuclear genetic and geographic distance in *C. guizhouensis* (Fig. S3). In addition, a U test showed that *N*_ST_ was not significantly greater than *G*_ST_, suggesting that there was no distinct phylogeographic structure of cpDNA in *C. guizhouensis*. However, obvious genetic structure was detected in *C. guizhouensis*. The UPGMA clustering of microsatellite data showed the 11 populations to comprise two clades (I and II) (Fig. S4). Populations in Guizhou and Guangxi province (except for population XL) were grouped into Clade I, while six populations in Yunnan province and population XL from Guangxi province were grouped into Clade II. The existence of the two clades was also supported by the Structure analysis (Fig. 5A), PCoA (Fig. 5B) and haplotype distributions (Fig. 1). The population XL is situated at the border of the two clades geographically and contains genetic components from both, suggesting this location as an area of admixture (gene flow) between the two clades.

Phylogenetic relationships of haplotypes of cpDNA and nDNA were reconstructed using Bayesian and Median Joining methods. Although monophyly of *C. guizhouensis* haplotypes from the four phylogenies was strongly supported by Bayesian inference, the phylogenetic relationships of haplotypes were not well resolved (Fig. S1). Nevertheless, network analysis provided a better resolution of the phylogenetic relationships of haplotypes (Fig. 2). Parsimony network analysis (except for the nuclear gene *F3H*) all suggested that there are two star-like evolutionary units separately dominating two lineages. The assumed two ancestral haplotypes were distributed in the inner nodes of the reticulate evolutionary diagrams at a high frequency. The derived haplotypes were distributed around the ancestral haplotypes with 1–3 mutational steps, showing a star-like topology, which indicated a recent rapid divergence. The topology of the haplotypes from the nuclear gene *F3H* was highly reticulate because eight recombination events had occurred (Fig. 2D).

Meanwhile, the BEAST-derived trees were consistent with the network of four DNA sequences (Fig. 3). Haplotypes were separated into two groups with an ancestral haplotype at the inner node of each group. All divergence times within *C. guizhouensis* estimated by BEAST occurred during the Middle-Upper Pleistocene, indicating that this species was the product of a recent rapid divergence, which is consistent with the former conclusion that the extant cycad species are not much older than 12 million years^14^. Previous studies have proposed that climatic oscillations in the Pleistocene had significant effects on the genetic diversity of many extant species^4^,^5^,^6^,^7^. However, different scales and frequencies of glaciations occurred in different regions in the Pleistocene^1^. In Europe, the Pleistocene ice sheet was evident during major glaciations^49^. In contrast, a relatively mild Quaternary climate occurred in China^3^. In general, the relatively high altitudes in the southwest mountains of China were deeply influenced by glaciations^50^,^51^, while the lower slopes or valleys were not affected during the cooler periods^52^. Therefore, we suggest that the relatively mild Pleistocene climate in China contributed to the survival and divergence of *C. guizhouensis* during that period.

Additionally, exploring the historical demography of a species can help in understanding its ancient evolutionary environment^9^. The glacial and interglacial climatic fluctuations have had profound influences on some plant species. To respond to the glaciation, most plants shifted latitude or elevation of their ranges^8^. Distinct demographics were detected in different plant taxa as a result of glacial and interglacial periods. Gymnosperm species, such as *Taxus wallichiana*^53^, *Cycas revoluta*, and *Cycas taitungensis*^54^, have experienced population expansions during the most recent glacial period, while *Cycas debaoensis*^27^, *Cycas simplicipinna*^9^ and *Cycas multipinnata*^10^ have experienced population contractions. In this study, four cpDNA, three nuclear genes and 13 microsatellites generated inconsistent results for the demography of *C. guizhouensis*. For cpDNA, neutrality tests and mismatch analysis showed that *C. guizhouensis* did not experience a recent population expansion (Table S4; Fig. S2A), while the Bayesian Skyline Plot revealed a recent population expansion (over 25–4 Ka years) in this species (Fig. 4A). For the three nuclear genes, *GTP* and *PHYP* indicated that *C. guizhouensis* experienced a recent population expansion; however, a recent population decline was detected by the nuclear gene *F3H* (Table S4; Fig. S2B–D; Fig. 4B–D). In addition, bottleneck analysis (Table 3) based on microsatellites showed that populations of *C. guizhouensis* have not experienced a recent bottleneck event, but these populations experienced a historical reduction in population size. Normally, different genes and markers are subjected to different selective pressures, which may generate the above inconsistency.

Marine Isotope Stages (MIS), based on oscillations in oxygen-18 values of benthic foraminifera, are timescales that are now widely used to express warm and cool periods in the Quaternary^55^. The odd-numbered troughs are warm or interglacial intervals, while even-numbered peaks are cold or glacial intervals^55^. Based on the Bayesian Skyline Plot results, *C. guizhouensis* was believed to have experienced an expansion during MIS1, except for the nuclear gene *F3H* (Fig. 4). *Cycas guizhouensis* had a population expansion after the post-glacial period (10 Ka-Present). As a result, Pleistocene climatic oscillations had no noticeable effect on the demography of *C. guizhouensis*. Alternatively, the warmer climate during the post-glacial period (MIS1) promoted the expansion of this species.

Information on the genetic diversity, genetic structure, and demographic history of rare and endangered species can aid the development of appropriate management strategies for their conservation. One goal for the conservation of threatened plants is to preserve their genetic diversity^56^. Our study detected relatively higher levels of genetic diversity in *C. guizhouensis* than some other *Cycas* species. However, massive illegal digging of *C. guizhouensis* for trading or ornaments has jeopardized the existing populations of this species and its effective population size. For example, some populations that were sampled in Xiao’s study, such as LP, have since been extirpated^19^. An effective population size greater than 100 is suggested to prevent inbreeding depression and a Ne greater than 1,000 is suggested to maintain the evolutionary potential of a population in perpetuity^57^. In contrast, the estimated Ne in populations of *C. guizhouensis* was less than 100 (especially less than 50), except for population LL (Table 2). Therefore, we suggest that protection zones or plots in the distribution areas of *C. guizhouensis* be established to protect the habitat for this species. In our study, two genetic clusters were detected in *C. guizhouensis*. Thus, these two genetic clusters could be managed as two evolutionary units and be given the highest priority protection. The demography of this species and network of its haplotypes indicated that there has been a recent expansion. Therefore, protection of the species in its natural habitat is required (*in situ*). Moreover, *ex situ* conservation is a viable auxiliary conservation approach for protecting this species by collecting seeds or seedling individuals from different populations which possess key sources of genetic diversity to remote adaptive areas. In addition, we suggest that more efforts should be made to raise awareness of conservation to local farmers and that a prohibition on deforestation in *Cycas* distribution areas be implemented. Taken together, these measures could protect *C. guizhouensis* from extinction.

## Methods

### Sampling and genotyping

A total of 209 individuals were collected from 11 populations of *C. guizhouensis* in southwestern Guizhou, northwestern Guangxi and eastern Yunnan province, China. Young and healthy leaves were dried in silica gel immediately after collection. All samples were stored in Kunming Institute of Botany, Chinese Academy of Sciences, China. Within the 209 samples, 10 individuals from each population were randomly selected for chloroplast and nuclear DNA sequencing, while all of the 209 individuals were used for the microsatellite study. Information on each sampling location and the number of individuals from each population that were used in the DNA sequence and microsatellite analyses are presented in Table S1 and Fig. 1, respectively. Among the 11 populations, seven populations are the same population examined in Xiao’s study and the same population codes are used^19^. Several of the populations examined in Xiao’s study are not included in this study because individuals from these populations (LP, MZ, TX, WF and XY) could not be detected. Fortunately, there were other populations of *C. guizhouensis* that were used in this study (AL, LW, XL and SP).

Genomic DNA was extracted from dried leaves using the modified CTAB method^58^. We chose four cpDNA intergenic spacers, *psb*A-*trn*H^32^, *trn*L-*trn*F^34^, *psb*M-*trn*D^33^ and *trn*L-*trn*T^34^ and three nuclear genes, *GTP*^35^, *PHYP* and *F3H* for complete analysis after preliminary screening from universal chloroplast and nuclear primers. The seven pairs of fragments were amplified for the most polymorphic sites of the 110 individuals. PCR amplification procedures were the same as those used in *C. simplicipinna*^9^. All PCR products were sequenced in both directions with the same primers for the amplification reactions, using an ABI 3770 automated sequencer at Shanghai Meiji Biological Medicine and Technology Co Ltd. All sequences were deposited in GenBank with the accession numbers KT824878-KT824962.

We screened microsatellite loci from recently developed nuclear microsatellites in *Cycas*^36^,^37^,^38^,^39^,^40^,^41^,^42^. PCR amplification was conducted with the same protocol as used in *C. simplicipinna*^9^. After preliminary screening microsatellite loci for *C. guizhouensis*, the selected microsatellite loci were stained with fluorescent dye at the 5′ end, their PCR products were separated and visualized using an ABI 3770 automated sequencer, and their profiles were read with GeneMapper software. An individual was declared null (nonamplifying) at a locus and was treated as missing data after two or more amplification failures. Finally, we chose 13 polymorphic microsatellite loci (Cha02^36^, Cha08^36^, Cy-TaiEST-SSR11^37^, E001^38^, E004^38^, Cpz26^39^, HL08^40^, CY232^41^, Cha-estssr01^42^, Cha-estssr02^42^, Cha-estssr04^42^, Cha-estssr03^42^, Cha05^36^) for *C. guizhouensis* after calculating polymorphism indices.

### Data analysis

#### Data analysis of DNA sequences

Sequences were edited and assembled using SeqMan. Multiple alignments of DNA sequences were performed manually and subsequently adjusted in Bioedit, version 7.0.4.1^59^. We combined the four cpDNA regions and performed a congruency test in PAUP* 4.0b10^60^. The testing results showed a significant rate of homogeneity (P = 1, >0.5), suggesting that there was a high degree of homogeneity between the four cpDNA regions. The combined cpDNA sequences were therefore used in following analyses. For three nuclear genes, heterozygous sites were identified by overlapping peaks in chromatograms. We resolved the nuclear sequences by applying the algorithms of PHASE^61^,^62^ in the software package DnaSP, version 5.0^63^. The phased nuclear sequences were used in following analyses. Maps were drawn using the software ArcGIS version 10.2 (http://desktop.arcgis.com).

We first used DnaSP, version 5.0^63^ to detect recombination in nuclear genes. Haplotypes were inferred from aligned DNA sequences by DnaSP, version 5.0^63^. Genetic diversity was estimated by calculating *Nei’s* nucleotide diversity (*P*i) and haplotype diversity (*H*d) indices using DnaSP, version 5.0^63^. We calculated within-population gene diversity (*H*_S_), gene diversity in total populations (*H*_T_)^64^, and two measures of population differentiation, *G*_ST_ and *N*_ST_, according to the methods described by Pons & Petit^65^ using the Permut, 1.0 (http://www.pierroton.inra.fr/genetics/labo/Software/Permut). We used the program Arlequin, version 3.11^66^ to conduct an analysis of molecular variance (AMOVA)^67^ and to estimate the genetic variation that was assigned within and among populations.

Phylogenetic relationships among cpDNA and nDNA haplotypes of *C. guizhouensis* were inferred using Bayesian methods implemented in MrBayes, version 3.1.2^68^. We chose *Cycas edentata* and *Cycas rumphii* as outgroups. The degree of relatedness among cpDNA and nDNA haplotypes was also estimated using Network, version 4.2.0.1^69^. In network analysis, indels were treated as single mutational events.

We used the evolutionary rates that had previously been estimated for seed plants to be 1.01 × 10^−9^ and 5.1−7.0 × 10^−9  ^70 mutations per site per year for synonymous sites to estimate the coalescent time of haplotypes for cpDNA and nDNA, respectively. Substitution models for cpDNA and nDNA were separately tested with MEGA, version 5^71^. We used BEAST, version 1.6.1^72^, to estimate the time of divergence using a strict molecular clock and the HKY model. The HKY + I model was used for the gene *F3H*. The BEAST program was also used to create a Bayesian Skyline Plot to infer the historical demography of *C. guizhouensis*. Posterior estimates of the mutation rate and time of divergence were obtained by Markov Chain Monte Carlo (MCMC) analysis. The analysis was run for 10^8^ iterations with a burn-in of 10^4^ under a strict clock and the HKY model or HKY + I model. Log parameters were sampled every 10,000 iterations. The analysis ran three times under the condition that all parameters were unchanged. The ESS parameter was found to exceed 200 by TRACER, version 1.5^73^, which suggested that there was acceptable mixing and sufficient sampling. Next, the three times running log files and tree files were combined, respectively. The combined log and tree files were used to construct a Bayesian skyline plot in TRACER, version 1.5^73^. We used a pairwise mismatch distribution to test for population expansion in DnaSP, version 5.0^63^, to further investigate the demography of the species. In addition, the signatures of demographic change were examined with neutrality tests, including Tajima’s *D*, Fu and Li’s D^*^ and F^*^ and Fu’s *F*_S_^44^, using DnaSP, version 5.0^63^. The sum-of-squared deviations (SSD) and raggedness index as well as their *P*-values were calculated with the software Arlequin, version 3.11^66^.

#### Data analysis of SSR markers

Dataset editing and formatting were performed in GenAlEx, version 6.3^74^. Genetic diversity indices, including the number of alleles (*N*_A_), private alleles (*A*_P_), effective number of alleles (*A*_E_), expected heterozygosity (*H*_E_), observed heterozygosity (*H*_O_), information index (*I*), fixation index (*F*) and percentage of polymorphic loci (*PPB*), were calculated using GenAlEx, version 6.3^74^, and POPGENE, version 1.32^75^, with mutual correction. Allelic richness (*A*_R_) was estimated with FSTAT, version 1.2^76^. The differentiation index *F*_ST_ between pairs of populations was computed with Arlequin, version 3.11^66^. Isolation by distance (IBD) was tested by performing Mantel tests in GenAlEx, version 6.3^74^ on the correlation of F_ST_/(1 − F_ST_) with geographic distance for all pairs of populations. F_ST_/(1 − F_ST_) was calculated with Genepop, version 4.1.4^77^. Gene flow between pairs of populations was estimated according to Wright’s principles *N*m = (1 − *F*_ST_)/4*F*_ST_^26^. Tests for departure from Hardy-Weinberg equilibrium (HWE) were performed in each locus and each population as well as a globally unified population using Genepop, version 4.1.4^77^.

The genetic structure of sampled populations was estimated by unweighted pair group mean analysis (UPGMA) using TEPGA, version 1.3^78^, with 5,000 permutations. A Bayesian analysis of population structure on the SSR data was conducted with STRUCTURE, version 2.2^79^. The combination of an admixture and a correlated-allele frequencies model was used for the analysis. The simulation was run with values of K from 1 to 20 and repeated 20 times for each set. Each run included a burn-in of 1 × 10^5^ iterations and 1 × 10^5^ subsequent MCMC steps. The best-fit number of groupings was evaluated using *Δ*K and the log-likelihood value by STRUCTURE HARVESTER, version 0.6.8^80^. Based on genetic distances among microsatellite phenotypes, an individual-based principal coordinate analysis (PCoA) was visualized with MVSP, version 3.12^81^.

The effective population sizes of each population were estimated in the program LDNe at three levels of the lowest allele frequency (0.01, 0.02, 0.05) with a 95% confidence interval^82^. We tested the bottleneck effect at the population level to explore population demography using different models and testing methods implemented in BOTTLENECK, version 1.2.02^83^. A stepwise mutation model (SMM) and a two-phased model (TPM) were chosen. Under the two models, the standardized differences test was removed from this study because this test is typically only used when at least twenty polymorphic loci are available. Two other methods (Sign tests and Wilcoxon tests) were applied in the analyses. We also used a mode shift model^84^ to test for bottlenecks in each population. These methods implemented in BOTTLENECK are most powerful when bottlenecks are severe and recent. These methods have low power unless the decline is greater than 90%^84^. Moreover, we further investigated a genetic bottleneck using the Garza-Williamson index (also called M-ratio, the ratio of number of alleles to range in allele size)^85^. The index was calculated by Arlequin, version 3.11^66^. When seven or more loci are analysed, the Garza-Williamson index is lower than the critical *M*c value of 0.68, a value obtained by simulations based on the empirical data in bottlenecked populations, which suggests a reduction in population size^66^,^85^. The Garza-Williamson index is more powerful for detecting genetic bottlenecks if the bottleneck lasted several generations or if the population made a rapid demographic recovery^86^. All figures were modified using Photoshop (Adobe Corporation, California, America). All statistical analyses were conducted under an open source environment. Methods were conducted in accordance with approved guidelines.

## Additional Information

**How to cite this article**: Feng, X. *et al*. Middle-Upper Pleistocene climate changes shaped the divergence and demography of *Cycas guizhouensis* (Cycadaceae): Evidence from DNA sequences and microsatellite markers. *Sci. Rep.* **6**, 27368; doi: 10.1038/srep27368 (2016).

## Supplementary Material

Supplementary Information

## Figures and Tables

**Table 1:** Analysis of molecular variance (AMOVA) based on DNA sequences and microsatellites for populations of *C. guizhouensis*, note: ^***^ *P* < 0.001.

**Marker**	**Source of variation**	**d.f.**	**Sum of squares**	**Variance components**	**Percentage of variation (%)**	***F***_**ST**_
cpDNA	Among populations	10	105.545	1.01172	69.82	0.698^***^
	Within populations	99	43.300	0.43737	30.18	
*GTP*	Among populations	10	6.809	0.02942	24.11	0.241^***^
	Within populations	209	19.350	0.09258	75.89	
*PHYP*	Among populations	10	21.818	0.09805	30.75	0.308^***^
	Within populations	209	46.150	0.22081	69.25	
*F3H*	Among populations	10	88.264	0.36458	19.20	0.192^***^
	Within populations	209	320.750	1.53469	80.80	
SSR	Among populations	10	197.779	0.44772	13.80	0.138^***^
	Within populations	407	1138.037	2.79616	86.20	

**Table 2:** Genetic diversity within populations of *C. guizhouensis*, note: *N*_T_, total number of alleles; *N*_P_, private alleles; *A*_R_, allelic richness; *N*_A_, number of alleles; *A*_E_, the effective number of alleles; *I*, Shannon’s information index; *H*_O_, observed heterozygosity; *H*_E_, expected heterozygosity; UHE, Nei’s unbiased heterozygosity; *F*, fixation index; *PPB*, the percentage of polymorphic loci; Ne, effective population size.

**Population**	***N***_**T**_	***N***_**P**_	***A***_**R**_	***N***_**A**_	***A***_**E**_	***I***	***H***_**O**_	***H***_**E**_	**UHE**	***F***	***PPB*****(%)**	**Ne**
AL	43	1	2.963	3.308	2.248	0.717	0.285	0.372	0.382	0.159	84.62	8
YP	33	0	2.255	2.538	1.680	0.543	0.250	0.328	0.336	0.163	84.62	13.8
LW	51	6	3.305	3.923	2.390	0.786	0.300	0.402	0.412	0.204	92.31	9.8
LL	57	6	3.530	4.385	2.753	0.770	0.281	0.362	0.371	0.246	76.92	981
XL	44	5	3.299	3.385	2.054	0.739	0.273	0.394	0.413	0.249	84.62	12.2
SZ	54	3	3.662	4.154	2.560	0.720	0.204	0.331	0.339	0.203	84.62	48.8
LX	67	3	4.292	5.154	3.118	1.003	0.331	0.463	0.475	0.243	92.31	21.7
ML	65	5	3.985	5.000	2.758	0.984	0.427	0.483	0.495	0.102	92.31	4.9
KY	65	6	4.001	5.000	2.674	0.947	0.323	0.453	0.464	0.212	92.31	34.2
JS	71	7	4.420	5.462	2.872	1.023	0.333	0.474	0.488	0.251	92.31	33.5
SP	76	7	4.799	5.846	3.757	1.175	0.415	0.546	0.560	0.214	92.31	51.9
Mean	57	4.46	3.656	4.378	2.624	0.855	0.311	0.419	0.431	0.204	88.11	110.9

**Table 3:** Bottleneck analysis using microsatellite loci from 11 populations of *C. guizhouensis*, note: *P* is test for heterozygosity excess, ^*^ *P* < 0.05, significantly different; ^**^ *P* < 0.01, highly significantly different.

**Population**	**T.P.M**		**S.M.M**		**Mode shift**	**Garza-Williamson**
	**Sign test**	**Wilcoxon test**	**Sign test**	**Wilcoxon test**		**index**
AL	0.007^**^	0.021^*^	0.122	0.080	L	0.433
YP	0.124	0.021^*^	0.297	0.376	L	0.359
LW	0.048^*^	0.058	0.460	0.787	L	0.414
LL	0.260	0.216	0.529	0.946	L	0.478
XL	0.141	0.146	0.473	0.787	L	0.455
SZ	0.038^*^	0.007^**^	0.298	0.191	L	0.424
LX	0.318	0.736	0.115	0.244	L	0.374
ML	0.308	0.168	0.524	0.946	L	0.426
KY	0.322	0.273	0.276	0.588	L	0.417
JS	0.556	0.636	0.119	0.340	L	0.436
SP	0.055	0.001^**^	0.302	0.273	L	0.363
