# Supplementary material for: Middle-Upper Pleistocene climate changes shaped the divergence and demography of Cycas guizhouensis (Cycadaceae): Evidence from DNA sequences and microsatellite markers
Source: Sci Rep. 2016 Jun 7;6:27368. doi: 10.1038/srep27368 (PMC4895228; doi:10.1038/srep27368)
Supplement: Supplementary Information [file srep27368-s1.pdf]

Title: Middle-Upper Pleistocene climate changes shaped the divergence and demography of *Cycas guizhouensis* (Cycadaceae): Evidence from DNA sequences and microsatellite markers

Authors: Xiuyan Feng, Ying Zheng & Xun Gong

Table S1. Details of sample locations, sample sizes (n) surveyed for DNA sequences and microsatellites of *C. guizhouensis*.

Population Code	Population	Latitude N°	Longitude E°	Altitude m	Individuals for DNA sequences/microsatellites
AL	Anlong, Guizhou	24.965	105.261	500	10/20
YP	Yangping, Xingyi, Guizhou	24.938	104.994	930	10/20
LW	Luowan, Xingyi, Guizhou	24.681	104.686	880	10/20
LL	Longlin, Guangxi	24.665	104.889	970	10/20
XL	Xilin, Guangxi	24.606	104.611	860	10/11
SZ	Shizong, Yunnan	24.652	104.248	1360	10/20
LX	Luxi, Yunnan	24.452	103.975	1160	10/20
ML	Mile, Yunnan	24.181	103.632	1430	10/20
KY	Kaiyuan, Yunnan	23.829	103.180	1400	10/20
JS	Jianshui, Yunnan	23.400	102.880	1320	10/18
SP	Shiping, Yunnan	23.720	102.500	1400	10/20
Total	11				110/209

Table S2. The composition of haplotypes, haplotype diversity (*H*d) and nucleotide diversity (*P*i) surveyed for combined cpDNA and nuclear genes of *C. guizhouensis*.

Population code	cpDNA (10)			*GTP* (20)			*PHYP* (20)			*F3H* (20)		
	Haplotypes (No.)	*H*d	*P*i × 10^3	Haplotypes(No.)	*H*d	*P*i × 10^3	Haplotypes(No.)	*H*d	*P*i × 10^3	Haplotypes(No.)	*H*d	*P*i × 10^3
AL	guiH1(10)	0	0	guiG1(20)	0	0	guiP1(3) guiP2(17)	0.268	0.27	guiF1(12) F2(1) F3(1) F4(2) F5(1) F6(2) F7(1)	0.642	2.79
YP	guiH1(10)	0	0	guiG1(20)	0	0	guiP1(5) guiP2(15)	0.395	0.39	guiF1(2) F6(18)	0.189	0.75
LW	guiH1(10)	0	0	guiG1(20)	0	0	guiP1(5) guiP2(15)	0.395	0.39	guiF1(12) F4(2) F6(6)	0.568	1.84
LL	guiH1(10)	0	0	guiG1(16) guiG2(4)	0.337	0.59	guiP1(8) guiP2(12)	0.505	0.50	guiF1(10) F4(3) F6(7)	0.637	1.96
XL	guiH1(2) guiH2(5) guiH3(1) guiH4(2)	0.733	0.54	guiG1(19) guiG3(1)	0.100	0.52	guiP1(9) guiP2(10) guiP3(1)	0.574	0.62	guiF1(8) F4(4) F6(1) F8(4) F9(1) F10(2)	0.784	5.68
SZ	guiH2(6) guiH5(4)	0.533	0.18	guiG1(20)	0	0	guiP1(18) guiP2(1) guiP4(1)	0.195	0.20	guiF4(1) F5(14) F6(3) F11(1) F12(1)	0.505	2.43
LX	guiH6(8) guiH7(2)	0.356	0.36	guiG1(20)	0	0	guiP1(19) guiP2(1)	0.100	0.10	guiF4(9) F5(1) F8(3) F13(3) F14(2) F15(2)	0.768	7.84
ML	guiH4(10)	0	0	guiG1(20)	0	0	guiP1(15) guiP2(3) guiP4(2)	0.426	0.46	guiF1(4) F2(1) F4(4) F5(1) F6(1) F8(1) F15(3) F16(3) F17(2)	0.900	4.96
KY	guiH8(8) guiH9(2)	0.356	0.24	guiG1(20)	0	0	guiP1(11) guiP2(5) guiP5(3) guiP6(1)	0.642	0.76	guiF2(2) F4(2) F8(4) F18(5) F19(1) F20(4) F21(1) F22(1)	0.874	6.99
JS	guiH2(9) guiH6(1)	0.2	0.27	guiG1(20)	0	0	guiP1(19) guiP4(1)	0.100	0.10	guiF1(2) F4(1) F5(12) F6(2) F23(2) F24(1)	0.637	3.29
SP	guiH2(2) guiH10(3) guiH11(3) guiH12(2)	0.822	1.63	guiG1(11) guiG4(1) guiG5(6) guiG6(1) guiG7(1)	0.632	2.44	guiP1(10) guiP2(3) guiP7(2) guiP8(1) guiP9(3) guiP10(1)	0.726	1.05	guiF1(2) F2(1) F4(1) F5(3) F16(1) F17(1) F25(2) F26-F29(1) F30(3) F31(1) F32(1)	0.958	6.06
Total	12	0.794	0.91	7	0.123	0.42	10	0.555	0.62	32	0.870	4.93

Table S3. Genetic diversity, differentiation parameters for the combined cpDNA sequences and nuclear genes in all populations of *C. guizhouensis*.

Marker	Hs	H_T	G_{ST}	N_{ST}
cpDNA	0.273	0.841	0.676	0.698
GTP	0.097	0.125	0.223	0.241
PHYP	0.393	0.571	0.311	0.307
F3H	0.678	0.902	0.248	0.192

Table S4. Parameters of neutrality tests and mismatch analyses based on cpDNA and nuclear genes of *C. guizhouensis*, note: **, $P < 0.01$; ***, $P < 0.001$.

Marker	Tajima'D	Fu and Li'D^*	Fu and Li'F^*	Fu's Fs	SSD	raggedness
cpDNA	0.74126	1.18632	1.22583	1.659	0.04352	0.23852
GTP	-1.67165	-1.75178	-2.05712	-6.269**	0.00753	0.11759
PHYP	-1.32224	-2.07520	-2.15827	-5.505**	0.03506	0.30727
F3H	-0.19361	1.40253	0.90810	-10.910***	0.07958	0.21545

Table S5. *P*-value of Hardy-Weinberg equilibrium tests for 11 populations of *C. guizhouensis*, note: -, Monomorphic; *, $P < 0.05$; **, $P < 0.01$; ***, $P < 0.001$.

Population	Cha02	Cha08	Cy-TaiEST-SSR11	E001	E004	Cpz26	HL08	CY232	Cha-estssr01	Cha-estssr02	Cha-estssr04	Cha-estssr03	Cha05	All loci
AL	0.002**	0.000***	1.000^{ns}	-	1.000^{ns}	0.544^{ns}	-	0.129^{ns}	0.000***	-	0.214^{ns}	0.280^{ns}	-	0.000***
YP	0.000***	0.000***	1.000^{ns}	-	1.000^{ns}	0.039*	-	0.002**	0.093	-	0.149^{ns}	1.000^{ns}	-	0.000***
LW	0.034*	0.000***	1.000^{ns}	0.026*	0.800^{ns}	0.009**	-	0.654^{ns}	0.000***	1.000^{ns}	1.000^{ns}	0.647^{ns}	-	0.000***
LL	0.017*	0.001**	-	-	1.000^{ns}	0.182^{ns}	-	1.000^{ns}	0.000***	0.026*	1.000^{ns}	0.528^{ns}	0.026*	0.000***
XL	0.173^{ns}	0.022*	-	-	0.406^{ns}	0.008**	-	0.143^{ns}	0.000***	0.023*	1.000^{ns}	0.215^{ns}	-	0.000***
SZ	0.013*	0.000***	-	1.000^{ns}	0.468^{ns}	0.041*	1.000^{ns}	0.004**	0.000***	1.000^{ns}	1.000^{ns}	-	-	0.000***
LX	0.035*	0.000***	-	0.003**	0.440^{ns}	0.052^{ns}	0.105^{ns}	0.198^{ns}	0.000***	0.000***	0.136^{ns}	-	-	0.000***
ML	0.001**	0.224^{ns}	0.633^{ns}	0.012*	0.156^{ns}	0.004**	1.000^{ns}	0.339^{ns}	0.000***	0.000***	0.018*	-	-	0.000***
KY	0.225^{ns}	0.000***	-	1.000^{ns}	0.076^{ns}	0.284^{ns}	1.000^{ns}	0.032*	0.000***	0.000***	0.619^{ns}	1.000^{ns}	-	0.000***
JS	0.000***	0.001**	0.169^{ns}	0.124^{ns}	0.000***	0.371^{ns}	1.000^{ns}	0.004**	0.000***	0.267^{ns}	0.036*	-	-	0.000***
SP	0.434^{ns}	0.017*	0.041*	0.208^{ns}	0.000***	0.656^{ns}	1.000^{ns}	0.054^{ns}	0.000***	0.026*	1.000^{ns}	0.544^{ns}	-	0.000***
All pop.	0.000***	0.000***	0.541^{ns}	0.001**	0.000***	0.000***	0.972^{ns}	0.000***	0.000***	0.000***	0.233^{ns}	0.832^{ns}	0.000***	0.000***

Table S6. Estimates of gene flow (*N*m) between pairs of *C. guizhouensis* populations.

Population	AL	YP	LW	LL	XL	SZ	LX	ML	KY	JS	SP
AL	0.000										
YP	1.620	0.000									
LW	2.354	1.187	0.000								
LL	2.328	0.893	12.096	0.000							
XL	0.937	0.591	1.345	1.224	0.000						
SZ	1.758	0.833	1.150	1.052	0.786	0.000					
LX	2.176	1.048	1.755	1.321	1.464	6.716	0.000				
ML	1.124	0.805	1.046	0.824	1.578	1.675	4.050	0.000			
KY	1.638	0.965	1.785	1.537	2.163	2.534	4.966	4.250	0.000		
JS	1.676	0.956	1.532	1.245	1.484	2.578	13.486	2.746	3.955	0.000	
SP	1.143	0.910	1.532	1.038	1.986	1.335	3.740	4.478	3.895	3.043	0.000

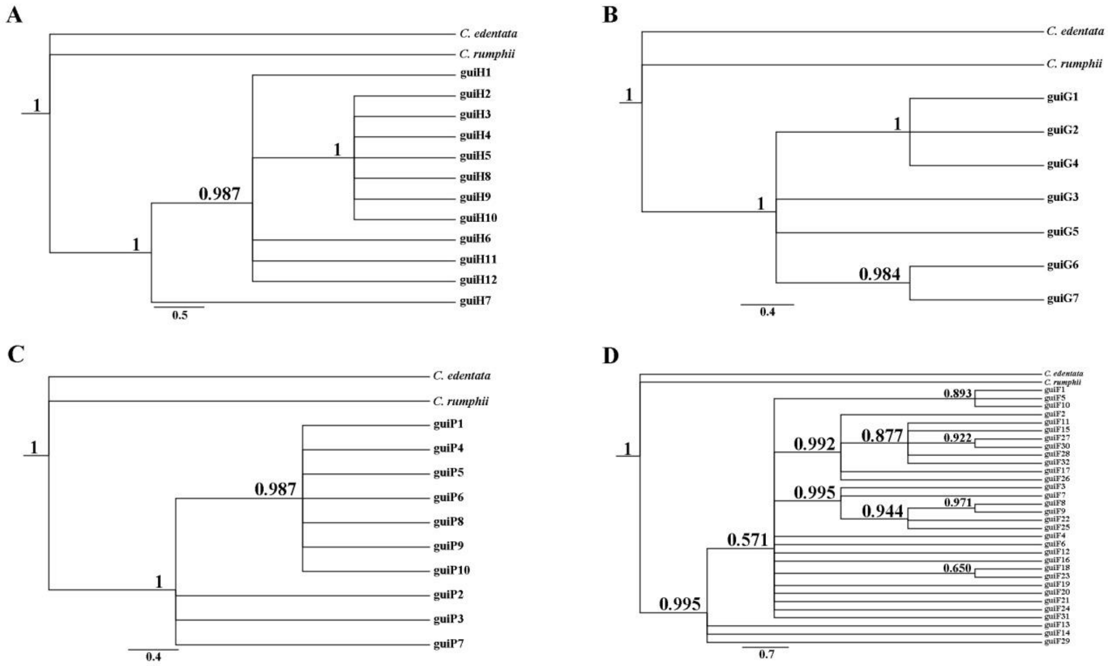

Figure S1. Bayesian tree based on the haplotypes of cpDNA (A), *GTP* (B), *PHYP* (C) and *F3H* (D). *Cycas edentata* and *Cycas rumphii* were used as outgroups. The number on each branch indicates the posterior probability (PP).

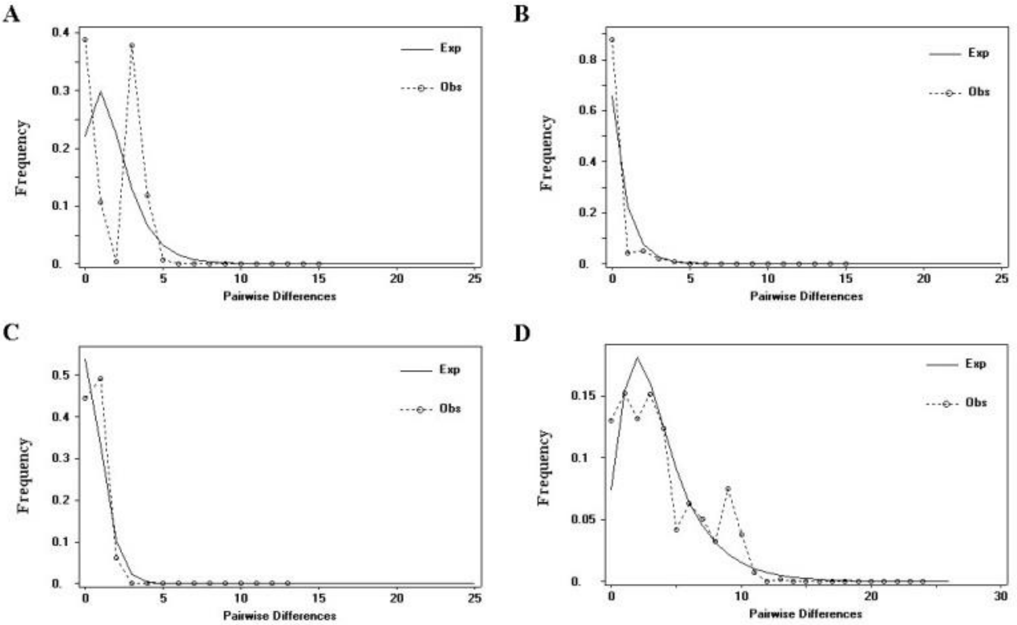

Figure S2. Mismatch distribution of cpDNA (A) and the nuclear genes *GTP* (B), *PHYP* (C) and *F3H* (D) haplotypes based on pairwise sequence differences against the frequency of occurrence for *C. guizhouensis*.

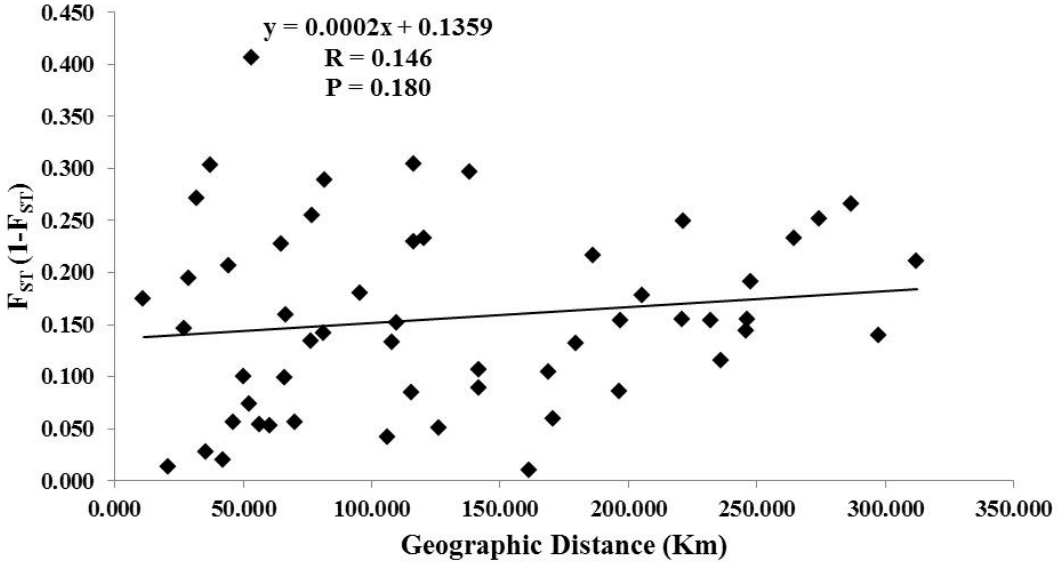

Figure S3. Figure plot of geographical distance against genetic distance for 11 populations of *C. guizhouensis*.

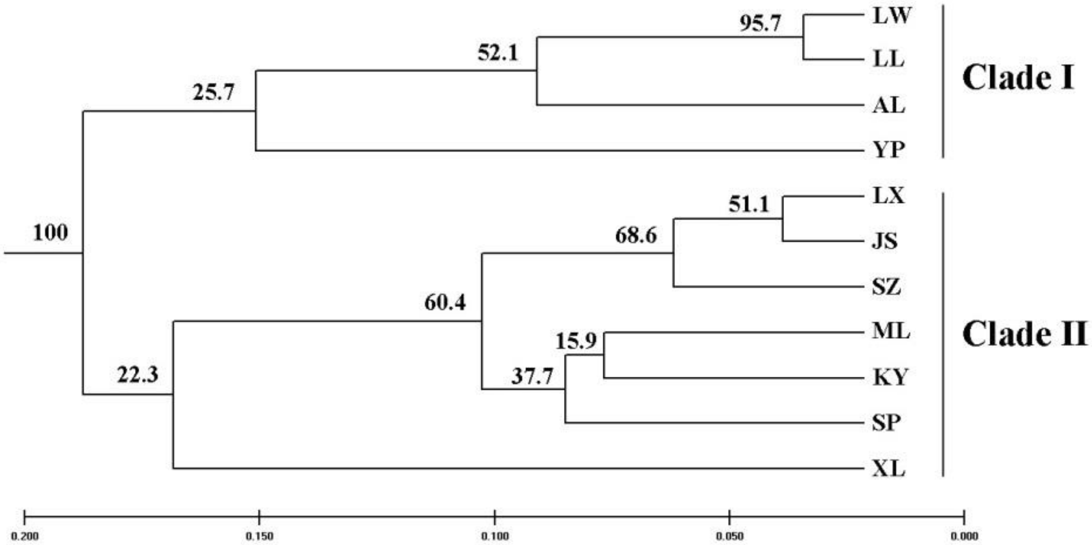

Figure S4. An unweighted pair-group method with arithmetic averages (UPGMA) phenogram of 11 populations of *C. guizhouensis* based on microsatellites. Numbers on branches indicate bootstrap values from 5,000 replicates.
